# The Value of Religion and Spirituality in the work and Personal Recovery of peer Support Specialists: an Exploratory Study in Israel

**DOI:** 10.1007/s10943-025-02409-0

**Published:** 2025-08-11

**Authors:** Ofra Walter, Batel Hazan-Liran

**Affiliations:** Tel Hai Academic College Israel, Qiryat Shemona, Israel

**Keywords:** Peer support, Spirituality, Mental health recovery, Belief in God, Higher power

## Abstract

We examined the role of spirituality in the recovery process in mental health care in Israel in the context of the October 2023 war, specifically as this role is perceived by peer support specialists. Peer support, based on principles of mutual connection, experiential knowledge, and empathy, focuses on empowering individuals and promoting the recovery process. Spirituality is a significant tool in this process. We administered questionnaires to a sample of 34 peer support specialists in Israel to understand their perceptions of the role of spirituality. Our findings showed a complex relationship between belief in a higher power and recovery, with some participants reporting feelings of guilt that hindered healing. The impact of spiritual experiences remained stable over time, with many participants maintaining their belief systems. The study highlights the challenges of integrating spirituality into peer support in Israel, particularly because of the different cultural and religious dynamics of religious and secular populations.

**Supplementary Information:**

The online version contains supplementary material available at 10.1007/s10943-025-02409-0.

## Introduction

Israel has made significant progress in adopting recovery-oriented approaches and rehabilitative care in mental illness over the past two decades. This shift has impacted the integration of individuals with mental health challenges into society and reduced the number of psychiatric hospitalizations (Aviram et al., 2023). The relatively new field of peer support, whereby individuals with a history of mental health challenges provide services to others facing similar struggles (Davidson et al., 2012), is one such approach. It is grounded in principles of mutual relationships, experiential knowledge, authentic empathy and validation (Mead & MacNeil, 2006), respect, shared responsibility, mutual agreement on the nature of assistance (Borkman et al., 2020), and mutual support (Repper & Carter, 2011). Models developed globally emphasize recovery, boundaries, the use of natural language, support, self-disclosure, and ethics (Barker & Maguire, 2017). While the recognition of the role of peer support and the value of experiential knowledge is steadily increasing, its practical application remains limited in Israel (Aviram et al., 2023).

Spirituality plays a significant role in enhancing peer support across populations. Spiritual well-being fosters coping strategies and strengthens the overall recovery process, as evidenced by multiple studies focusing on different cultural contexts and recovery frameworks (e.g., Lee, 2007; Maturlu, 2024). The October 7, 2024, massacre, Israel's subsequent invasion of Gaza, and the ongoing, albeit limited, violent conflict on Israel's northern border have had a profound impact on Israeli society. We argued that within this context, spirituality would emerge as especially salient to the efficacy of peer support. To test our argument, in an exploratory study, we examined 34 Israeli peer specialists’ perceptions of the role of spirituality in the recovery process for both their clients and themselves in the context of the October 2023 war.

## Literature Review

### Peer Support and its Role in Recovery

Peer support has increasingly become a critical component in addiction and mental health recovery systems. Rooted in the idea of mutual aid, the peer support model allows individuals to provide support to others with similar experiences, thereby fostering resilience, community building, and empowerment (White & Evans, 2015). Peer support workers, also known as recovery coaches or peer recovery support specialists, draw on their personal knowledge of recovery to offer emotional support, share practical strategies, and provide guidance through the challenges of the recovery process (Davidson et al., 2012). By relating to others through shared experiences, peer support specialists create an environment of trust and understanding that is often difficult to achieve through traditional clinical interventions alone (White & Evans, 2015).

The integration of peer support within recovery models has been particularly beneficial in supporting individuals who may be at risk of relapse or who face significant barriers to accessing ongoing treatment (Davidson et al., 2012; Granfield & Cloud, 2001). The benefits of peer support in recovery are well documented across a range of settings, including addiction recovery and mental health care (White & Evans, 2015). Peer support specialists also assist individuals in navigating complex systems of care, such as accessing housing, employment, and medical services, which are crucial for long-term stability and recovery (Granfield & Cloud, 2001). Importantly, the shift toward peer support has contributed to the de-stigmatization of mental health issues, fostering an environment where individuals feel empowered to share their experiences without fear of judgment (Granfield & Cloud, 2001; Repper & Carter, 2011). As peer support continues to grow in importance, ongoing research and careful program evaluation will be essential to ensure it complements and enhances traditional clinical models, contributing to more integrated and effective recovery systems (Granfield & Cloud, 2001).

### Spirituality and Religiosity in Peer Support

Spirituality and religiosity are two distinct but interrelated concepts with significant roles in mental health. Spirituality is often understood as a broad, personal experience of seeking meaning, connection with the transcendent, and self-transcendence. It is not confined to formal religious practices but can be expressed individually, in experiences of meaning-making, inner peace, purpose, transcendence, and connection to something greater than oneself (Koenig et al., 2012; Lee, 2007).

In contrast, religiosity involves adherence to organized religious practices, doctrines, and communal rituals. It is typically structured around specific beliefs, practices, and teachings that are followed by a religious community. Religiosity is often institutionalized and includes participation in formal religious activities, such as worship, prayer, and communal gatherings (Lee, 2007). While religiosity can provide a sense of belonging, identity, and purpose, it can also create stress when individuals experience conflict between their personal beliefs and the religious doctrines to which they adhere (Koenig et al., 2012; Maturlu, 2024).

The relationship between spirituality, religiosity, and mental health has been the subject of extensive research. Studies highlight the positive effects of spirituality on recovery outcomes, with spirituality often seen as a coping mechanism that provides individuals with a sense of purpose and community, particularly in faith-based settings (Dyani & Kelley, 2022). Individuals who engage in spiritual practices or have a strong sense of spiritual meaning often experience less anxiety, depression, and stress (Bovero et al., 2019; Zarzycka et al., 2019). Studies highlight the positive effects of spirituality on recovery outcomes, with spirituality often seen as a coping mechanism that provides individuals with a sense of purpose and community, particularly in faith-based settings (Dyani & Kelley, 2022). For example, participants in a recovery program reported that spirituality provided essential coping mechanisms, enhancing their resilience and ability to navigate challenges (He & Petrakis, 2023). The integration of spirituality into recovery practices, particularly in church-based interventions, has been found to improve recovery outcomes and reduce psychiatric symptoms (Rogers & Stanford, 2015).

Neurobiological research supports these findings, showing spiritual practices like mindfulness and meditation can enhance emotional regulation and foster prosocial behaviors, particularly through areas of the brain such as the dorsolateral prefrontal cortex and anterior cingulate cortex (Beerse et al., 2020; Wheeler et al., 2017).

Religiosity, while often linked to positive mental health outcomes, can have more complex effects. On the one hand, positive religious coping, such as seeking guidance or solace in faith in times of distress, is generally associated with better mental health outcomes (Duñó et al., 2020; Grover et al., 2021). On the other hand, negative religious coping, such as feelings of divine punishment or abandonment, can lead to increased psychological distress and exacerbate mental health symptoms (King et al., 2017).

Mental health professionals are increasingly recognizing the importance of addressing the spiritual and religious needs of their patients. Spirituality can serve as a powerful coping mechanism, and understanding how patients use their spiritual beliefs to navigate distress can enhance therapeutic interventions (Vieten & Scammell, 2015). Meanwhile, religious practices can provide patients with a sense of community, social support, and a framework for dealing with life's challenges, positively impacting their mental health (King, 2019; Koenig et al., 2012). However, some psychiatrists are hesitant to explore spiritual matters with patients, because of biases or a preference for biological models of mental illness (King, 2019). Despite these challenges, incorporating spiritual assessments into treatment plans is increasingly recognized as an essential aspect of providing holistic care that respects the cultural and individual needs of patients.

Spirituality and religiosity play a significant role in recovery processes facilitated by peer support. Rogers and Stanford (2015) argued peer support specialists frequently incorporate their spiritual beliefs into their practice, and this can strengthen the support they offer. Peer-led groups that integrate spirituality are associated with improved recovery outcomes and a reduction in psychiatric symptoms. However, spirituality can also have adverse implications, such as fostering feelings of self-blame or guilt, complicating the recovery process (Uota, 2012).

Spiritual well-being has been shown to mediate the relationship between peer support and mental health outcomes. A study on Indonesian migrant workers found peer support positively influenced mental health through spiritual well-being and coping strategies (Ghufron et al., 2024). A study on American Indian peers emphasized that recovery is inherently a spiritual process, highlighting themes of connection and belonging within peer support settings (Dyani & Kelley, 2022).

Many peer support specialists consider spirituality to be an essential component of their practice, enhancing their ability to empathize with and connect to individuals in recovery. Research indicates that spiritual practices, such as prayer and meditation, can be beneficial for both peer support specialists and those they support, fostering resilience and emotional well-being (Saiz et al., 2021). Moreover, the spiritual transformation experienced by their clients often reinforces peer support specialists’ own recovery journey, enhancing their leadership role within the peer support context (Glickman et al., 2006).

Overall, the relationship between spirituality and recovery is complex, with both positive and negative experiences reported, highlighting the need for a nuanced understanding of the role of spirituality (Uota, 2012). While spirituality can enhance recovery experiences, it is crucial to recognize the potential for negative impacts, underscoring the importance of careful integration of spiritual practices in peer support settings.

### Challenges and Opportunities in Integrating Spirituality/Religiosity into Peer Support

The integration of spirituality and religiosity into peer support presents both significant challenges and promising opportunities. While the potential benefits include enhanced emotional support and improved recovery outcomes, various ethical and practical concerns must be addressed to ensure effective implementation.

A key challenge in integrating spirituality into peer support is the potential for conflicts of belief. Peer support specialists may encounter tensions between their own spiritual or religious views and those of the individuals they support, complicating the therapeutic relationship (Parada, 2022). Ethical considerations, such as maintaining boundaries and respecting diverse belief systems, are crucial in this context. Failure to respect these boundaries can lead to moral dilemmas and disrupt the support process (Currier et al., 2023). A specific concern is the possibility of peer support specialists unintentionally imposing their religious beliefs on clients; this may undermine the supportive and non-judgmental nature of peer relationships (Dean et al., 2008). Additionally, peer support specialists who are experiencing their own spiritual crises may find it challenging to provide effective support to others (Wu et al., 2020).

Despite these challenges, the integration of spirituality into peer support has several potential benefits. Research suggests it can foster resilience, provide a sense of purpose, and offer emotional support, all of which can enhance recovery outcomes (Rogers & Stanford, 2015). Moreover, peer support specialists themselves may experience professional and personal growth through spiritual exploration, which can further enrich their ability to support others (Currier et al., 2023). Finally, spirituality has the potential to enhance empathy and deepen understanding in peer support relationships, leading to improved rapport and a more effective support experience (Parada, 2022).

### Peer Support Specialist Culture and Religiosity in the Israeli Context

The cultural context of spirituality and religiosity significantly influences peer support practices. Spirituality is often intertwined with cultural identity, affecting how individuals perceive and engage with their beliefs (Weaver, 2008). Different religious backgrounds lead to varied practices and beliefs, impacting peer support dynamics. For instance, some cultures may emphasize communal support, while others may focus on individual spirituality (Abo-Zena and Akef, 2024). Understanding these variations is crucial for peer support specialists to provide assistance.

In Israel, the Community Mental Health Rehabilitation Law has resulted in the development of community-based rehabilitation services for individuals with mental health challenges. This includes training courses for peer support specialists, formally supported by the Israeli Ministry of Health since 2005 (Grundman et al., 2021). The Ministry's support for peer support began with the launch of a training course for rehabilitation workers with lived experience (Aviram et al., 2023).

In the Israeli context, religious and cultural values shape the experiences of peer support. Many peer support specialists draw from their Jewish heritage, which often includes an emphasis on community solidarity, compassion, and healing. These values can enhance the therapeutic impact of peer support, fostering deeper connections between peer specialists and those they assist and helping supporters navigate the complex interaction between religiosity and secular mental health practices. Israel’s unique cultural context is characterized by its diverse religious and ethnic communities, including Jewish, Arab, and immigrant communities, each with its own distinct cultural norms and values (Sagi & Nachtomy, 2019). Even particular groups can show considerable within-group differences. For example, the interplay between secular Jews (Hilonim) and ultra-Orthodox Jews (Haredim) creates a complex landscape for social services and peer support initiatives. Peer support specialists must navigate these cultural differences to be effective; this requires cultural competence and sensitivity to varying religious practices and beliefs (Ritblatt & Rosental, 2018). Tensions between religious and secular populations can hinder collaboration in peer support settings, as differing worldviews may lead to misunderstandings (Ben-Porat & Feniger, 2012). An ongoing cultural conflict will impact the willingness of individuals from different backgrounds to engage in peer support, as trust and acceptance are crucial for effective communication (Kosiorek et al., 2024).

## The Present Study

Based on the literature, we formulated the following research questions on the value of spirituality to peer support specialists:

1. How do peer support specialists perceive spirituality as a tool in their work?
2. How do peer support specialists perceive spirituality in the context of their personal recovery process?
3. Have the perceptions of peer support specialists changed over the years?

## Method

We undertook exploratory research to answer our research questions and gain an in-depth understanding of the role of spirituality in the recovery process of Israeli peer support specialists. Exploratory research is particularly appropriate when existing literature is limited, and the objective is to uncover patterns, generate insights, and lay the foundation for future research (Stebbins, 2001). In this context, the present study was warranted by the scarcity of empirical data on how certified peer specialists in Israel experience and integrate spirituality into their personal recovery and professional practices. While precise figures are not publicly available, it is estimated that several dozen individuals have completed formal training and are currently active as peer support specialists within Israel’s mental health system (Moran, 2018; Walter & Hazan-Liran, 2023). The Israeli Ministry of Health has supported the development of this role through pilot programs and national initiatives. A total of 34 certified peer specialists responded and participated in the study.

### Participants

The study included 34 Jewish peer support specialists in Israel. They had all taken specialized training to serve as peer support specialists. On average, they had worked as peer support specialists for 3.39 years (SD = 3.36), with years of experience ranging from 1 to 14. They all reported mental health and substance abuse issues or psychiatric hospitalizations. Table 1 shows the average and standard deviation by health issue and the type of recovery program participants were involved in. Given the sensitive nature of the topics discussed—particularly spirituality, trauma, and recovery in the context of ongoing conflict—ethical considerations were a priority throughout the study. Participants were informed of their right to withdraw at any time, confidentiality was assured, and efforts were made to ensure that no questions were phrased in a manner that could trigger re-traumatization. The study received ethical approval from the relevant institutional review board, and care was taken to approach the subject matter with sensitivity and respect.

**Table 1:** Participant Demographic Characteristics (N = 34): Averages and Standard Deviations of Assistance by Peer Support Specialist Population and Program Type

Variables	Category	n	M	SD	Min	Max
**All Sample**		34	2.93	1.38	1	5
**Sample Type**	Mental health issues	30	3.03	1.38	1	5
	Mental health and substance issues	4	2.20	1.30	1	4
**Program Type**	Psychiatric hospitalization	2	1.90	0.42	1.6	2.2
	Community integration	21	2.97	1.45	1	5
	Family counseling and support centers	1	3.00	-	3	3
	Other	10	3.04	1.43	1	5

## Measures

Participants responded to a 68-item survey with closed questions on demographic characteristics (race, ethnicity, gender identity), experiences providing peer support (certification status, current work setting), and past and current experiences with spirituality and religion (see the Appendix for the complete survey). The survey items were derived either from established measures of spirituality and religion or were specifically developed for this study, based on insights from a prior study of peer support specialists’ experiences using spirituality and religion in their work [BLINDED FOR REVIEW].

To address the role of faith and spirituality in recovery (research question 1), a study-specific item was developed on the role of faith and spirituality in the recovery process, rated by participants on a 5-point Likert scale (1 = very negative role, 5 = very positive role).

Items testing spirituality, religiousness, and personal beliefs (question 2) included such questions as: ‘To what extent does any connection to a spiritual being help you to get through hard times?’ Responses were rated on a 5-point scale (1 = not at all; 5 = extremely).

To determine participants’ evolving experiences with faith and spirituality, a series of questions compared past versus current experiences (research question 3). These items were original to the study and were rated by participants on a 1–6 scale (1 = strongly disagree; 6 = strongly agree); for example, ‘I feel I am loved by God’ A study-specific item assessed whether experiences with faith and spirituality had changed over time, rated on a 3-point scale (1 = yes, a lot; 2 = yes, a little; 3 = no). They were asked: ‘In what ways have your experiences with faith/spirituality changed over time?’ Participants were also asked to describe their current experience with faith and spirituality, with responses rated on a 5-point Likert scale (1 = very negative; 5 = very positive). Several items examined various aspects of current faith and spirituality experiences. For example, a question asked how welcomed participants felt in a faith or spiritual community, rated on a 5-point scale (1 = not at all; 5 = extremely). Reliability analysis for the total scale (α = .89).

## Data Analysis

In a variety of statistical analyses, we examined the relationships and differences between variables for spirituality, religious beliefs, and recovery processes. We used Spearman's correlations to assess the strength and direction of the association between the frequency of discussing spiritual or religious matters with their clients and the perceived ability of these discussions to support the recovery process. We used Mann–Whitney tests to explore significant differences between groups (secular Jews and ultra-Orthodox Jews), based on their belief in God or a higher power, spirituality, and practices such as yoga or meditation. Correlations were calculated between variables such as experiences with religious figures, the role of belief/spirituality in the recovery process, and the perceived impact of spirituality on coping. Finally, we used Wilcoxon tests to compare participants' perceptions of their relationship with God or a higher power and related emotions, such as love and anger, between the past and the present. These statistical tests allowed a detailed exploration of how spiritual beliefs and experiences intersected with the participants' own recovery journeys.

## Results

We conducted a Spearman’s correlation test to determine whether there was a statistically significant correlation between the frequency with which participants discussed religious or spiritual matters with peers as part of their work as peer support specialists and the perceived support of these discussions in the recovery process. We found a significant negative correlation, r(32) = -0.372, p = 0.030. In other words, the higher the frequency with which participants discussed religious or spiritual matters with peers as part of their work, the lower the perceived support of these discussions to the recovery process.

We conducted several Mann–Whitney analyses to determine whether there were statistically significant differences between participant groups (secular, religious) based on the belief that a connection with a spiritual entity aids in coping, As shown in Table 2, we only found a significant statistical difference based on belief in God or a higher power. Specifically, those who believed in God or a higher power perceived a connection with a spiritual entity as more helpful in coping than those who did not.

**Table 2:** Differences in the Belief that a Connection with a Spiritual Entity Assists in Coping

Variable	Category	n	M	SD	Z
Belief in God or a higher power	No	14	1.79	0.91	
	Yes	20	3.73	1.05	**Z = −3.92, p < .001**
I am spiritual	No	20	2.80	1.39	
	Yes	14	3.11	1.39	Z = −0.76, p = 0.450
Yoga/meditation practice	No	30	2.99	1.40	
	Yes	4	2.50	1.25	Z = −0.75, p = 0.3452
None of the above	No	26	3.13	1.24	
	Yes	8	2.28	1.69	Z = −1.43, p = 0.154

We conducted several Mann–Whitney analyses to determine whether there were statistically significant differences based on participants’ perceptions of their current experience with faith or spirituality. As shown in Table 3, we only found a significant statistical difference based on belief in God or a higher power. Specifically, those who believed in God or a higher power rated their current experience with faith or spirituality higher than those who did not.

**Table 3:** Differences Based on Current Experiences of Faith or Spirituality

Variable	Category	n	M	SD	Z
Belief in God or a higher power	No	14	3.57	1.09	
	Yes	20	4.30	0.66	**Z = −2.07, p = 0.039**
I am spiritual	No	20	3.95	0.89	
	Yes	14	4.07	1.00	Z = −0.48, p = 0.632
Yoga/meditation practice	No	30	3.97	0.93	
	Yes	4	4.25	0.96	Z = −0.56, p = 0.573
None of the above	No	26	4.12	0.77	
	Yes	8	3.63	1.30	Z = −0.94, p = 0.346

We used Mann–Whitney analyses to examine whether there were statistically significant differences between groups (secular, religious) based on perceptions of changes over the years in the personal experience of faith/spirituality. As shown in Table 4, we did not find any statistically significant differences.

**Table 4:** Differences Based on the Perception of Changes over the Years in Personal Experience of Faith/Spirituality

Variable	Category	n	M	SD	Z
Belief in God or a higher power	No	14	1.71	0.73	
	Yes	20	1.65	0.75	Z = −0.31, p = 0.760
I am spiritual	No	20	1.75	0.72	
	Yes	14	1.57	0.76	Z = −0.80, p = 0.422
Yoga/meditation practice	No	30	1.70	0.75	
	Yes	4	1.50	0.58	Z = −0.41, p = 0.683
None of the above	No	26	1.69	0.74	
	Yes	8	1.63	0.74	Z = −0.22, p = 0.824

We used Mann–Whitney analyses to look for statistically significant differences based on perceptions of the role of faith/spirituality in the personal recovery process. As shown in Table 5, a significant statistical difference only appeared for belief in God or a higher power. Specifically, those who believed in God or a higher power rated the role of faith/spirituality in the personal recovery process lower than those who did not.

**Table 5:** Differences Based on the Perception of the Role of Faith/Spirituality in the Personal Recovery Process

Variable	Category	n	M	SD	Z
Belief in God or a higher power	No	14	2.43	1.02	
	Yes	20	1.65	0.81	**Z = −2.30, p = 0.022**
I am spiritual	No	20	1.90	1.07	
	Yes	14	2.07	0.83	Z = −0.85, p = 0.394
Yoga/meditation practice	No	30	2.03	1.00	
	Yes	4	1.50	0.58	Z = −1.02, p = 0.308
None of the above	No	26	1.85	0.83	
	Yes	8	2.38	1.30	Z = −0.99, p = 0.323

To examine whether there were statistically significant differences based on feelings of anger toward God/higher power in the past, we conducted several Mann–Whitney analyses. As Table 6 indicates, no statistically significant differences emerged.

**Table 6:** Differences Based on Feelings of Anger toward God/Higher Power in the Past

Variable	Category	n	M	SD	Z
Belief in God or a higher power	No	14	2.86	2.03	
	Yes	20	3.05	1.96	Z = −0.14, p = .885
I am spiritual	No	20	2.90	2.10	
	Yes	14	3.07	1.82	Z = −0.58, p = .562
Yoga/meditation practice	No	30	3.10	1.95	
	Yes	4	2.00	2.00	Z = −1.22, p = .223
None of the above	No	26	2.88	1.86	
	Yes	8	3.25	2.38	Z = −0.42, p = .674

To examine whether there were statistically significant differences based on the perception of anger toward God/higher power in the present, we conducted further Mann–Whitney analyses. As Table 7 shows, we did not find any statistically significant differences.

**Table 7:** Differences in Feelings of Anger toward God/Higher Power in the Present

Variable	Category	n	M	SD	Z
Belief in God or a higher power	No	14	1.71	1.38	
	Yes	20	1.85	1.39	Z = −0.32, p = .747
I am spiritual	No	20	1.50	1.00	
	Yes	14	2.21	1.72	Z = −1.71, p = .087
Yoga/meditation practice	No	30	1.90	1.42	
	Yes	4	1.00	0.00	Z = −1.60, p = .110
None of the above	No	26	1.77	1.27	
	Yes	8	1.88	1.73	Z = −0.07, p = .944

We conducted Spearman’s correlations to determine whether there were statistically significant correlations between the following variables: connection to a spiritual entity (God/higher power) as helping coping; negative and positive experiences with religious figures (e.g., spiritual leader); discussions of belief/spirituality in sessions with clients as supporting the healing process; and belief/spirituality as assisting in the participants’ own healing process. As Table 8 reveals, we found statistically significant correlations between several variables.

**Table 8:** Correlations Between Variables: Connection to God/Higher Power Helping Coping, Negative and Positive Experiences with Religious Figures, Discussions on Belief/Spirituality Supporting the Healing Process of Clients, and the Role of Belief/Spirituality in the Personal Healing Process

Variables	1	2	3	4
1. Connection to God/higher power helps coping				
2. Religious figures—negative experience	−0.556**			
3. Religious figures—positive experience	.389*	−0.092		
4. Discussions of belief/spirituality support the healing process	−0.363*	0.187	−0.309	
5. Belief/spirituality plays a role in the personal healing process	−0.553**	0.241	−0.061	0.338*

^*^p < .05 ** p < .01 *** p < .001

We found significant negative correlations between the perception that the connection to a spiritual entity (God/higher power) helps coping and negative experiences with religious figures, and between perceptions that discussions on belief/spirituality support clients’ healing process and perceptions of the role of belief/spirituality in the personal healing process. We also found a significant positive correlation between perception that the connection to a spiritual entity helps coping and positive experiences with religious figures. In other words, participants’ more positive perception of the connection to God/higher power as helping coping meant participants had fewer negative experiences with religious figures; more positive experiences with religious figures meant a lower perception of the contribution of discussions on belief/spirituality to the healing process and a lower perception of the role of belief/spirituality in the healing process.

Finally, there was a significant positive correlation between perceptions of discussions on belief/spirituality as supporting the healing process and perceptions of the value of belief/spirituality in the healing process. We did not find any other statistically significant correlations.

We conducted Wilcoxon tests to determine whether there were differences between the past and the present in participants’ understanding of God/higher power. Table 9 shows statistically significant differences in participants’ relationship with God/higher power, feelings of love from God/higher power, and anger toward God/higher power. In the present, there was more concern for the relationship with God/higher power, a feeling of less love from God/higher power, and more anger toward God/higher power than in the past. No differences were found for participation in religious rituals or the meaning of God/higher power in personal life.

**Table 9:** Differences Between Past and Present in Perceptions of God/Higher Power

Variable	Category	n	M	SD	Z
Concern for the relationship with God/higher power	Present	34	3.21	2.20	
	Past	34	2.35	1.92	**Z = -2.68, p = .007**
Feeling loved by God/higher power	Present	34	3.38	2.17	
	Past	34	4.06	2.27	**Z = -2.31, p = .021**
Participation in religious rituals is beneficial	Present	34	3.15	2.15	
	Past	34	3.21	1.89	Z = -0.44, p = .657
God/higher power has meaning in personal life	Present	34	4.12	2.17	
	Past	34	3.94	2.16	Z = -0.60, p = .545
Feelings of anger toward God/higher power	Present	34	2.97	1.96	
	Past	34	1.79	1.37	**Z = -3.31, p = .001**

## Discussion

Our first research question centered on peer support specialists’ perceptions of the use of spirituality in their work. The findings revealed a complex relationship between spirituality, belief in God or a higher power, and the recovery process in peer support contexts. Specifically, belief in God or a higher power was associated with a lower perceived role of spirituality in the recovery process. This is somewhat counterintuitive, as the literature generally argues spirituality can be a positive coping mechanism, providing emotional resilience, purpose, and emotional well-being (Lee, 2007; Maturlu, 2024). However, our findings suggest that for some individuals, the role of spirituality, specifically in the form of belief in a higher power, may not always align with beneficial recovery outcomes.

Several possible explanations may shed light on these findings. One possibility is that for individuals who perceive their belief in God or a higher power as an essential aspect of their recovery, spirituality may become linked to feelings of guilt, self-blame, or the sense that they are being punished for their illness (Martínez et al., 2023). In these cases, spirituality may create internal conflicts, as individuals may struggle with the perceived disconnect between their beliefs and their recovery, leading to heightened distress rather than alleviation. This is consistent with research showing that negative religious coping, such as feelings of divine abandonment or punishment, can exacerbate mental health symptoms (King et al., 2017). While we touched on negative religious coping (e.g., guilt or anger toward God), we did not deeply investigate how these specific experiences may hinder the recovery process. A more detailed exploration of such mechanisms could have provided a more nuanced and balanced view of spirituality's role in mental health. Understanding how negative spiritual experiences contribute to psychological distress is crucial, especially when spirituality is a central part of an individual’s worldview. This gap points to the need for future research that systematically examines how negative spiritual struggles may function as barriers to healing and asks what therapeutic approaches might address them (Exline et al., 2014).

In Israel, the lack of a clear connection between spirituality and the recovery process can be attributed to several factors rooted in the country’s cultural and religious dynamics. The ongoing tension between religious and secular populations creates a fragmented landscape for recovery. Secular Jews (Hilonim) tend to favor conventional, non-spiritual approaches to mental health, while religious Jews (Haredim) emphasize spirituality, community support, and faith-based healing (Sagi et al., 2019). This division affects the trust and mutual understanding necessary for effective peer support (Ben-Porat & Feniger, 2012).

Israel's lack of separation between religion and state further complicates the integration of spirituality into recovery programs. While spirituality may play a role in therapeutic practices in other countries, in Israel, state and religious influences can create conflicting expectations of its role in peer support (Ritblatt & Rosental, 2018). The national security situation, including ongoing wars, may shift the focus away from personal or spiritual healing toward collective survival, reducing the perceived relevance of spirituality in recovery (Kosiorek et al., 2024). Thus, the absence of a strong connection between spirituality and recovery in Israel is shaped by cultural divides, the intertwining of religion and state, and the broader sociopolitical context, all of which influence the effectiveness of peer support initiatives (Gerber-Epstein, 2014). Peer support specialists must navigate these complexities to address diverse needs in a multifaceted environment.

Our second question concerned peer support specialists’ perceptions of spirituality in the context of their personal recovery process. The role of spirituality in recovery has long been recognized as pivotal in promoting resilience and coping mechanisms (Dyani & Kelley, 2022). We added to the literature by considering the complexities introduced by cultural and religious diversity, as well as the context of the ongoing war. The findings suggest perceptions of spirituality may affect recovery in a region marked by ongoing conflict and trauma. Moreover, it seemed peer support specialists who believed in a higher power were not necessarily more spiritual than those who did not have similar beliefs and therefore did not always use it in their work. Arguably, for them, faith represented or symbolized mental illness.

Whether participants were religious or secular, spirituality was often integrated into their peer support. For many peer support specialists in Israel, spirituality especially in the form of Jewish beliefs in God or a higher power may serve as a core element of their coping strategies. However, frequent discussions of spirituality in peer support were negatively correlated with perceptions of the ability of spirituality to support recovery. Overly frequent discussions may re-trigger trauma or past emotional struggles, particularly in the context of war. Data for this study were collected during the war. The conflict has led to increased trauma, heightened anxiety, and distress, making spirituality a sensitive and sometimes fraught topic. In our sample, peer support specialists with negative experiences of God/higher power—often linked to feelings of anger or disillusionment—reported lower perceived benefits from spirituality discussions. This is consistent with the literature showing negative religious experiences can complicate the healing process (Kosiorek et al., 2024). In the context of war, such negative emotions may be amplified, as individuals may feel their spiritual beliefs have abandoned them or may struggle with feelings of betrayal by a higher power.

We found significant differences in how peer support specialists who believed in God or a higher power perceived the role of spirituality in coping. Those believing in a spiritual entity were more likely to report that spirituality helped them cope, aligning with findings showing belief in a higher power can be a key coping mechanism in recovery (Dyani & Kelley, 2022). However, spirituality alone may not significantly contribute to the recovery process. Specifically, those who believed in God or a higher power perceived their connection with a spiritual entity as more helpful in coping, but when rating the role of faith or spirituality in their personal recovery, they tended to assign it a lower value than those who did not believe in a higher power. This suggests that belief itself may not be a powerful driver of recovery.

The experience with significant spiritual figures—such as a religious leader or a meaningful spiritual connection—appeared to play a more significant role. Participants who engaged with them seemed to find more support and meaning in their recovery process than those who relied solely on belief in a higher power. The finding suggests recovery is likely more influenced by meaningful encounters with spiritual figures than by belief alone. It also emphasizes the importance of personalized and relational aspects of spirituality in recovery. Peer support specialists may benefit more from having tangible, meaningful spiritual connections, such as with mentors or religious leaders, than having a general belief system.

Our last question asked whether peer support specialists’ perceptions of spirituality changed over the years. The results revealed some important patterns in spiritual experiences and their effect on healing processes. The participants’ perception of spirituality remained largely unchanged. Those who previously believed in God continue to hold this belief, with no significant shifts in belief. Similarly, participants who harbored anger toward God in the past continued to feel the same way in the present. Such consistency could be indicative of a deep-seated relationship with spirituality that is resistant to change, even in the face of ongoing personal struggles. It may also reflect a coping mechanism, where spiritual beliefs act as an anchor during difficult times. Even when participants experienced anger or disillusionment, their faith itself remained intact, possibly because it provided a framework for understanding suffering and adversity. This could explain why anger toward God does not necessarily diminish over time; it may become an integrated part of one's spiritual identity.

Another significant finding was the increased level of anger participants felt toward God in the present, compared to the past. This heightened anger could be interpreted as a response to ongoing suffering or perceived abandonment. As previous research suggests, individuals feeling anger toward God often report a spiritual struggle, which can hinder their ability to rely on spirituality for support (Exline, 2019; Martínez et al., 2023). It is possible that anger toward God in the present may lead individuals to disengage from spirituality as a resource in their recovery. While participants may have relied on their spiritual beliefs in the past, the current emotional distance from God may prevent them from using spirituality as a tool for healing. The role of anger in this context should not be underestimated. Anger may serve as an emotional barrier, making it difficult for individuals to access the emotional support that spirituality typically offers. If anger toward God is a prominent emotion, it may lead to a form of spiritual disconnection that could impair an individual’s ability to find peace or solace, even in the presence of faith.

In the context of Israel, where religiosity is deeply ingrained in the culture, individuals who experience spiritual struggles—such as anger or disillusionment with God—may face additional social challenges. Israeli religiosity, which often emphasizes conformity to mainstream religious norms, may be less accepting of individuals who deviate from religious narratives, particularly those who experience doubts or anger toward God. This cultural pressure may exacerbate feelings of isolation, making it even more difficult for individuals to express or work through their spiritual struggles.

### Limitations and Future Research

The study had a few limitations. The sample size was small, and this may have impacted the generalizability of the findings. The small sample size was partly due to the relatively nascent development of the peer support specialist role in Israel; only a small number of individuals are officially designated as peer support specialists. Future research should include a larger, more diverse sample as the field continues to expand.

Second, relying on quantitative questionnaires limits the depth of the insights. Future research could incorporate qualitative interviews, direct observations, or triangulation to gain a richer understanding. Third, our sample included only Jewish peer support specialists, restricting exploration across Israel's diverse religious and ethnic communities. Although we widely disseminated the questionnaire, responses came exclusively from Jewish participants. Future studies should intentionally recruit more diverse samples to enhance generalizability across cultural contexts and conduct longitudinal research to better understand the evolving role of spirituality in recovery processes.

## Conclusion

The role of spirituality in mental health recovery depends on a range of personal, cultural, and psychological factors. The findings highlight the importance of tailoring peer support to meet the diverse needs of individuals, recognizing that for some, spiritual or religious frameworks may provide comfort and strength, while for others, they may serve as barriers, especially when linked to negative coping strategies or internalized stigma. In the Israeli context, where religious and secular populations coexist in a highly charged environment, particularly amidst ongoing war, cultural sensitivity in peer support becomes even more crucial. As the conflict deepens, peer support specialists must navigate not only the personal traumas of those they assist but also the broader cultural and spiritual tensions. Frequent discussions on spirituality may benefit those who find solace in religious practices, but for others, particularly those with negative associations with religious figures, such discussions may prove harmful. Thus, the integration of spirituality into peer support must be flexible; it must be responsive to the spiritual needs and emotional challenges of individuals.

The study has significant implications for the practice of peer support, particularly in contexts marked by cultural and religious diversity, as well as ongoing conflict. It highlights the need for a nuanced understanding of spirituality in recovery, emphasizing that belief in a higher power and spiritual practices are not universally beneficial. Instead, the impact of spirituality on recovery is highly individualized, influenced by personal experiences, cultural background, and emotional state. In environments like Israel, where religious and secular communities coexist amidst the pressures of war, peer support programs must be flexible and culturally sensitive. Practitioners should be mindful of the diverse ways spirituality can either support or hinder recovery, ensuring peer support strategies are tailored to meet the specific needs of each individual. This research underscores the importance of addressing both the spiritual and psychological dimensions of recovery, particularly during times of crisis, to foster a more effective and compassionate approach to peer support.

## Supplementary Information

Below is the link to the electronic supplementary material.

Supplementary file1 (DOCX 33 KB)
