# Supplementary material for: The Value of Religion and Spirituality in the work and Personal Recovery of peer Support Specialists: an Exploratory Study in Israel
Source: J Relig Health. 2025 Aug 11;65(1):554–72. doi: 10.1007/s10943-025-02409-0 (PMC12913310; doi:10.1007/s10943-025-02409-0)
Supplement: Supplementary file 1 — Supplementary file1 (DOCX 33 KB) [file 10943_2025_2409_MOESM1_ESM.docx]

**Appendix**

**Peer Support Specialist Survey**

A variety of programs in Israel offer training for individuals with lived experience in mental health recovery, leading to their recognition as certified peer support specialists or a similar form of certification.

**1. Are you considered a peer support specialist?**

- No
- Yes
- I received a similar certificate in another country.

**2. In what year did you complete the training and receive the certificate?**

**3. Have you ever been employed in a position that required you to be a peer support specialist?**

- No
- Yes

**4. Are you currently employed in a position that requires you to be a peer support specialist?**

- No
- Yes

**5. What type of population have you mainly worked with or are currently working with?**

- People with mental health issues
- People with both mental health and substance use issues
- People with substance use issues

**6. What type of program are you currently working in?**
(If you are not currently working as a peer support specialist, please select the type of program you most recently worked in. Choose the closest option if none are an exact match.)

- Psychiatric hospitalization
- Day clinics
- Community integration programs
- Family counseling and support centers
- Other (please specify): ___________

**7. Have you ever participated in a 12-step-oriented peer support group, such as Alcoholics Anonymous or similar groups?**

- No
- Yes

**8. What is your gender identity?**

- Male
- Female
- Other

**Belief/Spirituality**

We will ask you about your personal experiences regarding faith/spirituality throughout this survey. We understand that some of these questions may evoke challenging emotions or thoughts. Participation is voluntary, and if at any point you feel uncomfortable, we recommend discontinuing the survey.

**9. People experience faith/spirituality in many ways. Please check all statements that describe your approach to faith/spirituality:**

- I believe in God or a higher power.
- I consider myself spiritual, reflecting a connection to nature, others, or something beyond the physical world, but not necessarily involving God or a higher power.
- My connection to faith/spirituality is through practices such as yoga, meditation, or mindfulness.
- None of the above (please briefly explain): _______________

**10. Some people’s faith/spirituality is tied to a specific religion or identification. Which of the following best describes your religious affiliation (or lack thereof)?**

- Christian – Protestant
- Christian – Catholic
- Christian – Other (please specify): _____
- Muslim
- Jewish
- Atheist

**11. Please rate the extent to which you agree or disagree with the following statements:**
(Scale: 1 = Strongly Disagree to 6 = Strongly Agree)

| Item | Strongly Disagree (1) | Moderately Disagree (2) | Slightly Disagree (3) | Slightly Agree (4) | Moderately  Agree (5) | Strongly  Agree (6) |
| --- | --- | --- | --- | --- | --- | --- |
| 3. I used to feel loved by God or a higher power. |  |  |  |  |  |  |
| 4. I currently feel loved by God or a higher power. |  |  |  |  |  |  |
| 5. I used to find it helpful to participate in religious ceremonies.  6. I currently find it helpful to participate in religious ceremonies.  7. I used to think God or a higher power gave my life meaning.  8. I currently think God or a higher power gives my life meaning.  9. I used to feel angry at God or a higher power.  10. I currently feel angry at God or a higher power. |  |  |  |  |  |  |

**12. How would you generally describe your current experience with faith or spirituality in your life?**

- Very negative
- Negative
- Neither negative nor positive
- Positive
- Very positive

**13. Has your experience with faith/spirituality changed over the years?**

- Yes, significantly
- Yes, somewhat
- No

**14. In what way has your experience changed?**

- Much more negative now
- Slightly more negative now
- No change
- Slightly more positive now
- Much more positive now

**15. What role has faith/spirituality played in your recovery process?**

- Very positive role
- Positive role
- No role at all
- Negative role
- Very negative role

**16. Please rate the extent to which the following statements are true for you:**
(Scale: 1 = Not at all, 5 = Extremmelly)

Please rate the degree to which these questions apply to you

|  | Not at all (1) | A little (2) | A moderate amount (3) | Very much (4) | Extremely (5) |
| --- | --- | --- | --- | --- | --- |
| To what extent does any connection to a spiritual being help you to get through hard times? (1) |  |  |  |  |  |
| To what extent does any connection to a spiritual being help you to tolerate stress? (2) |  |  |  |  |  |
| To what extent does any connection to a spiritual being help you to understand others? (3) |  |  |  |  |  |
| To what extent does any connection to a spiritual being provide you with comfort / reassurance? (4) |  |  |  |  |  |
| To what extent do you feel welcomed and embraced in a faith or spiritual community? (5) |  |  |  |  |  |

**17. Please rate the extent to which you agree or disagree with the following statements:**
(Scale: 1 = Strongly Disagree, 6 = Strongly Agree)

|  | Strongly Disagree (1) | Moderately Disagree (2) | Slightly Disagree (3) | Slightly Agree (4) | Moderately Agree (5) | Strongly Agree (6) |
| --- | --- | --- | --- | --- | --- | --- |
| I currently have bad memories of past experiences with religion or religious people (1) |  |  |  |  |  |  |
| I have been treated well by people in faith communities because of my mental health issues. (2) |  |  |  |  |  |  |
| I have been told by religious people that not praying enough or having enough faith or punishment from God was the cause of my mental health issues. (3) |  |  |  |  |  |  |
| I have had disagreements with a family member or friend about religious issues (4) |  |  |  |  |  |  |
| I have had disagreements with something that your religion or church teaches. (5) |  |  |  |  |  |  |
| I feel lonely or different from others because of my religious/spiritual beliefs (6) |  |  |  |  |  |  |
| Mental health professionals have actively encouraged me to pursue my religious/spiritual beliefs (7) |  |  |  |  |  |  |
| Mental health professionals have actively supported me in pursuing my religious/spiritual beliefs, such as helping me search for information, obtain transportation, and attend events. (8) |  |  |  |  |  |  |

**Spirituality in Your Work as a Peer Support Specialist**

In this section, we are interested in understanding how spirituality is expressed in your work as a peer support specialist, and whether you are interested in acquiring further knowledge or guidance on integrating spirituality in this context. When referring to religion or spirituality here, we are not referring to practices such as yoga, mindfulness, or meditation. Please do not consider these when answering the following questions.

**18. How often do you talk about religious or spiritual issues with peers as part of your peer support work?**

- Never
- Rarely
- Sometimes
- Often
- Very often

**19. Do you typically initiate discussions about religion/spirituality, or do your peers usually bring it up first?**

- I often initiate the topic
- My peers usually bring it up
- I don’t discuss religious/spiritual topics with the peers I work with

**20. Would you like to talk more, less, or about the same as you currently do with your peers about faith/spirituality?**

- More
- Less
- About the same

**21. How easy or comfortable is it for you to talk about faith/spirituality with peers?**

- Very easy
- Moderately easy
- Somewhat easy
- Somewhat difficult
- Moderately difficult
- Very difficult

**22. Do you routinely ask about faith/spirituality during intake or other assessment processes with peers?**

- Yes
- No
- I am not involved in intake or assessment processes

**23. In the past year, have you offered a peer support group focused partially or entirely on religious/spiritual issues (excluding yoga, mindfulness, or meditation)?**

- Yes
- No
- I do not facilitate groups as part of my peer support work

**24. How often have you provided peers with information on faith/spirituality topics they expressed interest in?**

- Never
- Rarely
- Sometimes
- Often
- The peers I work with do not request such information

**25. Please describe how you have supported peers in accessing resources related to their faith/spiritual interests:**

**26. In your opinion, how much do discussions about faith/spirituality support the recovery process of your peers?**

- Very supportive
- Somewhat supportive
- Not supportive

**27. To what extent do you think discussions about faith/spirituality might be harmful to peers you support?**

- Not harmful
- Slightly harmful
- Moderately harmful
- Very harmful

**28. Please describe some ways in which it could be harmful:**

**29. Overall, how important is it for you to integrate conversations about faith/spirituality into your peer support work?**

- Not important
- Slightly important
- Moderately important
- Very important

**30. Please rate the extent to which you agree or disagree with the following statements:**
(Scale: 1 = Strongly Disagree, 6 = Strongly Agree)

|  | Strongly Disagree (1) | Moderately Disagree (2) | Slightly Disagree (3) | Slightly Agree (4) | Moderately Agree (5) | Strongly Agree (6) |
| --- | --- | --- | --- | --- | --- | --- |
| Faith/spirituality was important in my recovery, which is why I want to support my peers in this area if it is of interest to them. (1) |  |  |  |  |  |  |
| My supervisor encourages me to mention faith/spiritual issues with peers I am working with and support them if this is an area that is of interest to them. (2) |  |  |  |  |  |  |
| My agency/organization has a positive attitude toward discussions about faith/spirituality with my peers. (3) |  |  |  |  |  |  |
| I have clear guidelines on how I am able to discuss faith/spirituality matters with peers I work with. (4) |  |  |  |  |  |  |
| I have received training about how to discuss faith/spirituality topics with peers I am working with. (5) |  |  |  |  |  |  |
| I lack knowledge about faith/spiritual issues, which makes me uncomfortable talking to my peers about this issue. (6) |  |  |  |  |  |  |
| I have been explicitly told by a supervisor or someone else in my organization not to discuss matters of faith/spirituality with peers I am working with. (7) |  |  |  |  |  |  |
| I am worried about putting my faith/spiritual beliefs on others. (8) |  |  |  |  |  |  |
| I am concerned about discussing faith/spiritual matters with peers I work with because it might bring up some of their mental health issues (e.g., religious delusions; some peers get obsessed with religious issues). (9) |  |  |  |  |  |  |
| I am concerned that if I bring up faith/spirituality as a topic of discussion that the peers I work with might stop talking with me. (10) |  |  |  |  |  |  |
| The lack of program or agency guidelines about whether or how we should be talking about faith/spirituality leads me to be uncomfortable bringing it up. (11) |  |  |  |  |  |  |
| I am concerned that I might not be able to understand another peer’s religious/spiritual beliefs if they differ from my own. (12) |  |  |  |  |  |  |

**31. Did your peer support training include specific information on how to integrate faith/spirituality in your work with peers?**

- Yes
- No
- Don’t remember

**32. Has your supervisor ever provided guidance on how to integrate faith/spirituality into peer support or service delivery?**

- Yes
- No
- Don’t remember

**33. Do you have specific guidelines or a clear policy at your organization for how to talk about faith/spirituality with peers?**

- Yes
- No
- Don’t remember

**34. If yes, please briefly describe those guidelines:**

**35. To what extent would you be interested in training on best practices for integrating faith/spirituality in your peer support work?**

- Not at all interested
- Slightly interested
- Moderately interested
- Very interested

**36. Would you be interested in joining a peer community that discusses how faith and spirituality are integrated into peer support relationships?**

- Not at all interested
- Slightly interested
- Moderately interested
- Very interested

**37. Is there anything else you would like to share about faith/spirituality in the context of peer support work?**
